# Effect of a Fracture Liaison Service on the Rate of Subsequent Fracture Among Patients With a Fragility Fracture in the Norwegian Capture the Fracture Initiative (NoFRACT): A Trial Protocol

**DOI:** 10.1001/jamanetworkopen.2018.5701

**Published:** 2018-12-07

**Authors:** Camilla Andreasen, Lene B. Solberg, Trude Basso, Tove T. Borgen, Cecilie Dahl, Torbjørn Wisløff, Gunhild Hagen, Ellen M. Apalset, Jan-Erik Gjertsen, Wender Figved, Lars M. Hübschle, Jens M. Stutzer, Jan Elvenes, Ragnar M. Joakimsen, Unni Syversen, Erik F. Eriksen, Lars Nordsletten, Frede Frihagen, Tone K. Omsland, Åshild Bjørnerem

**Affiliations:** 1 Department of Orthopedic Surgery, University Hospital of North Norway, Tromsø, Norway; 2 Department of Clinical Medicine, Arctic University of Norway, Tromsø, Norway; 3 Division of Orthopedic Surgery, Oslo University Hospital, Oslo, Norway; 4 Department of Orthopedic Surgery, St Olav’s University Hospital, Trondheim, Norway; 5 Department of Rheumatology, Vestre Viken Hospital Trust, Hospital of Drammen, Drammen, Norway; 6 Department of Community Medicine and Global Health, Institute of Health and Society, University of Oslo, Oslo, Norway; 7 Department of Infectious Disease Epidemiology and Modelling, Norwegian Institute of Public Health, Oslo, Norway; 8 Department of Reviews and Health Technology Assessments, Norwegian Institute of Public Health, Oslo, Norway; 9 Bergen Group of Epidemiology and Biomarkers in Rheumatic Disease, Department of Rheumatology, Haukeland University Hospital, Bergen, Norway; 10 Department of Global Public Health and Primary Care, University of Bergen, Bergen, Norway; 11 Department of Orthopedic Surgery, Haukeland University Hospital, Bergen, Norway; 12 Department of Clinical Medicine, University of Bergen, Bergen, Norway; 13 Department of Orthopedic Surgery, Bærum Hospital, Vestre Viken Hospital Trust, Bærum, Norway; 14 Department of Orthopedic Surgery, Vestre Viken Hospital Trust, Hospital of Drammen, Drammen, Norway; 15 Department of Orthopedic Surgery, Møre and Romsdal Hospital Trust, Molde Hospital, Molde, Norway; 16 Department of Medicine, University Hospital of North Norway, Tromsø, Norway; 17 Department of Endocrinology, St Olav’s University Hospital, Trondheim, Norway; 18 Department of Clinical and Molecular Medicine, Norwegian University of Science and Technology, Trondheim, Norway; 19 Department of Endocrinology, Morbid Obesity and Preventive Medicine, Oslo University Hospital, Oslo, Norway; 20 Department of Clinical Medicine, University of Oslo, Oslo, Norway; 21 Department of Obstetrics and Gynecology, University Hospital of North Norway, Tromsø, Norway

## Abstract

**Importance:**

Fragility fracture is a major health issue because of the accompanying morbidity, mortality, and financial cost. Despite the high cost to society and personal cost to affected individuals, secondary fracture prevention is suboptimal in Norway, mainly because most patients with osteoporotic fractures do not receive treatment with antiosteoporotic drugs after fracture repair.

**Objectives:**

To improve secondary fracture prevention by introducing a standardized intervention program and to investigate the effect of the program on the rate of subsequent fractures.

**Design, Setting, and Participants:**

Trial protocol of the Norwegian Capture the Fracture Initiative (NoFRACT), an ongoing, stepped wedge cluster randomized clinical trial in 7 hospitals in Norway. The participating hospitals were cluster randomized to an intervention starting date: May 1, 2015; September 1, 2015; and January 1, 2016. Follow-up is through December 31, 2019. The outcome data were merged from national registries of women and men 50 years and older with a recent fragility fracture treated at 1 of the 7 hospitals.

**Discussion:**

The NoFRACT trial is intended to enroll 82 000 patients (intervention period, 26 000 patients; control period, 56 000 patients), of whom 23 578 are currently enrolled by January 2018. Interventions include a standardized program for identification, assessment, and treatment of osteoporosis in patients with a fragility fracture that is led by a trained coordinating nurse. The primary outcome is rate of subsequent fracture (per 10 000 person-years) based on national registry data. Outcomes before (2008-2015; control period) and after (2015-2019; intervention period) the intervention will be compared, and each hospital will act as its own control. Use of outcomes from national registry data means that all patients are included in the analysis regardless of whether they are exposed to the intervention (intention to treat). A sensitivity analysis with a transition window will be performed to mitigate possible within-cluster contamination.

**Results:**

Results are planned to be disseminated through publications in peer-reviewed journals and presented at local, national, and international conferences.

**Conclusions:**

By introducing a standardized intervention program for assessment and treatment of osteoporosis in patients with fragility fractures, we expect to document reduced rates of subsequent fractures and fracture-related mortality.

**Trial Registration:**

ClinicalTrials.gov Identifier: NCT02536898

## Key Points

**Question:**

What is the effect of a fracture liaison service on the rate of subsequent fractures?

**Findings:**

This trial protocol intends to use merged outcome data from national registers to include 82 000 women and men 50 years and older with a fragility fracture treated in 7 hospitals in Norway in a stepped wedge cluster randomized clinical trial introducing a standardized intervention program. The use of outcome data from national registers, which include all patients in the analysis regardless of whether they are exposed to the intervention (intention to treat), should ensure that outcomes are assessed in a standardized way.

**Meaning:**

The design of this trial is intended to overcome the ethical challenges associated with traditional randomized clinical trials and to generate new knowledge on how to improve the current standard of care.

## Teaser

This trial protocol of the Norwegian Capture the Fracture Initiative (NoFRACT) intends to use registry data from Norway and a stepped wedge cluster design to investigate the effect of a standardized intervention program on the rate of subsequent fracture among patients with a fragility fracture.

## Introduction

Among women, a prior fracture doubles the risk of a future fracture and multiple fractures increase the risk of future fractures up to 5 times.^1^ One-third of fractures occur within the first year and 75% of subsequent fractures after a hip fracture occur within 5 years.^2,3^ Hip and vertebral fractures are known to increase mortality, and new data suggest that other major osteoporotic fractures are also associated with a reduction in life expectancy.^4^ Treatment of patients with fragility fractures should therefore not only focus on the current fracture but also on preventing future fractures. Even though treatment for osteoporosis is readily available and can reduce the risk of future fractures by 20% to 50%,^5,6^ the assessment and treatment for osteoporosis after a fragility fracture has been suboptimal. Two Norwegian studies^7,8^ have demonstrated that only 15% of women and 4% of men were treated with antiosteoporotic drugs after a hip fracture,^7^ and 11% of women and 3% of men were treated with antiosteoporotic drugs the first year after a forearm fracture.^8^ These findings are consistent with those of studies from other countries where less than 20% of patients with a fragility fracture received treatment for osteoporosis.^9,10^

A fracture liaison services (FLS) model of care with a dedicated coordinator and a systematic approach to identify, assess, and treat patients with a fragility fracture for osteoporosis has been introduced in numerous places.^11,12^ The FLS programs have been shown to increase the referrals to bone mineral density (BMD) measurements using dual-energy x-ray absorptiometry for screening of osteoporosis.^12^ In a Swedish minimal FLS that was coordinated by medical secretaries, the proportion of patients receiving dual-energy x-ray absorptiometry evaluation increased from 8% to 40% and the treatment rate increased from 13% to 32%. Furthermore, individuals who received treatment had a 51% lower risk of subsequent fractures compared with those who did not receive treatment.^13^

Few researchers have studied subsequent fracture rates and mortality as outcomes in studies of the effect of FLS programs.^13,14,15,16,17,18,19,20^ In Australia, a 30% reduction in risk of any subsequent fracture and a 40% reduction in risk of major subsequent fractures were shown.^14^ In the Netherlands, a significant decrease in mortality of 33% was reported,^15^ and in England, a reduction around 20% was found.^16^ However, a Swedish study showed no effect of FLS on subsequent fracture rates or mortality, and an Australian study reported the effect of FLS on risk of subsequent fractures and no effect on mortality rates.^13,14^ Studies of FLS with subsequent fracture rates and mortality as outcomes are presented in Table 1. Although previous FLS studies are of great value, there has, to our knowledge, been no randomized clinical trial on FLS with fracture rates and mortality as outcomes. In a recent observational study,^21^ reduced mortality and subsequent fracture risk was shown in individuals who were recommended anti-osteoporotic drugs as part of an FLS program. The authors of that study proposed that traditional randomized clinical trials of FLS are unlikely to be performed given the ethical challenges of randomizing some individuals to less-than-recommended care. Moreover, the FLS program has not been evaluated on a public health scale, and more robust evidence is therefore needed.^11,22,23^

**Table 1.** Studies of FLS With Rates of Subsequent Fracture and Mortality as Outcomes

Source	Data Source	Intervention vs Control						
		Study Design	Patients, No.	Women, %	Mean Age, y	Absolute Fracture Rates, %	Rate of Subsequent Fracture Rate, HR (95% CI)	Mortality Rate, HR (95% CI)
Huntjens et al,^17^ 2014	*ICD-9* fracture codes, national obituary database (date of death)	FLS vs non-FLS at different hospitals; prospective design	1412 vs 1910	73 vs 70	71.1 vs 69.6	6.7 vs 6.8^a^	1-y follow-up: 0.84 (0.64-1.10); 2-y follow-up: 0.44 (0.25-0.79)	At 2 y: 0.65 (0.53-0.79)
Nakayama et al,^14^ 2016	Emergency department (fracture codes)	FLS vs non-FLS at different hospitals; prospective design, intention-to-treat approach	515 vs 416 (103 attended FLS)	75 vs 74	76.6 vs 75.0	12.2 vs 16.8	Any refracture, 3-y follow-up: 0.67 (0.47-0.95); major refracture, 3-y follow-up: 0.59 (0.39-0.90)	HR, 1.17^b^
Hawley et al,^16^ 2016^c^	*ICD-10* (hip fracture), Office for National Statistics (mortality)	Pre-FLS and post-FLS; before-after time series design	33 152	78	82.7	4.2	1.03 (0.85-1.26)	At 30 d: 0.80 (0.71-0.91); at 1 y: 0.84 (0.77-0.93)
Axelsson et al,^13^ 2016	*ICD-10*, Swedish Population Register (death information)	Pre-FLS vs post-FLS; prospective design with historic controls	2713 vs 2616	73 vs 74	76.1 vs 76.7	8.4 vs 8.3	0.95 (0.79-1.14)	0.88 (0.76-1.03)
Huntjens et al,^15^ 2011	*ICD-9*, national obituary database	Pre-FLS vs post-FLS	1920 vs 1335	75 vs 73	70.8 vs 71.9	9.9 vs 6.7	2-y follow-up: 0.65 (0.51-0.84)	At 2 y: 0.67 (0.55-0.81)
Van der Kallen et al,^18^ 2014	Diagnosis codes	FLS nonattendees vs FLS attendees; prospective design	220 vs 214	77 vs 79	74 vs 72	18.6 vs 6.5	2-y follow-up: 18.6 vs 6.5^d^	NA
Astrand et al,^19^ 2012	Questionnaire	Pre-FLS vs post-FLS; historic controls	306 vs 286	72 vs 76	NA	29 vs 18	6-y follow-up: 0.58 (0.39-0.89)	17 vs 12^a^^,^^d^
Lih et al,^20^ 2011	Not mentioned	Nonattendees vs attendees; MTF service; prospective controlled observational design	156 vs 246	75 vs 83	65.9 vs 66.4	19.7 vs 4.1	Median 38-mo follow-up: 5.3 (2.71-11.6)	NA

Abbreviations: FLS, fracture liaison service; HR, hazard ratio; *ICD-9, International Classification of Diseases, Ninth Revision*; *ICD-10, International Statistical Classification of Diseases and Related Health Problems, Tenth Revision*; MTF, minimal trauma fracture; NA, not applicable.

^a^ Nonsignificant.

^b^ The 95% CI was not provided in the original article.

^c^ Because of the study design, data are shown for 1 group.

^d^ Absolute rates because HRs and 95% CIs were not calculated.

When the Norwegian Capture the Fracture Initiative (NoFRACT) study was designed in 2014, there was no systematic routine or national guideline for the identification and treatment of patients with fragility fractures in Norway. Most patients were offered neither assessment nor treatment for osteoporosis. This gap in care motivated a large-scale evaluation of the effect of introducing a standardized secondary fracture prevention program on subsequent fracture rates and mortality in Norway. The NoFRACT study is, to our knowledge, the first FLS study with a stepped wedge cluster randomized clinical trial design, overcoming the ethical challenges of a traditional randomized clinical trial. The study may generate new knowledge and provide evidence on how to improve the current standard of care.

The study has aimed to assess the effect of introducing an FLS standardized intervention program for the treatment of osteoporosis in patients with a fragility fracture on subsequent fracture rates (per 10 000 person-years). The study has also assessed the effect of introducing an FLS standardized intervention program for treatment of osteoporosis in patients with a hip fracture on all-cause mortality after hip fracture.

## Methods

### Study Setting

This trial protocol followed the Standard Protocol Items: Recommendations for Interventional Trials (SPIRIT) reporting guideline. For largely unknown reasons, Norway is among the countries with the highest incidence rates of forearm and hip fractures worldwide.^24,25,26^ Each year, almost 10 000 Norwegians older than 50 years have a hip fracture, and the annual number of forearm fractures is estimated to be approximately 15 000.^25,27,28^ Hip fractures account for almost 20% of fragility fractures,^29^ implying a total of approximately 50 000 osteoporotic fractures annually in Norway. Despite a decline in age-standardized hip fracture rates between 1999 and 2013, the total number of hip fractures has increased owing to the aging population.^26,30,31^ The ongoing NoFRACT multicenter study is being conducted in the orthopedic departments at the following 7 hospitals in Norway: University Hospital of North Norway, Tromsø; St Olav’s University Hospital, Trondheim; Oslo University Hospital, Oslo; Haukeland University Hospital, Bergen; Molde Hospital, Molde; Drammen Hospital, Drammen; and Bærum Hospital, Bærum. These hospitals represent both smaller and larger hospitals and are geographically spread across Norway. The NoFRACT study has received approval from the Regional Committee for Medical and Health Research Ethics, Region for South Eastern Norway, for the merging of data from national registers and exemption from obtaining consent from the patients with fractures. All data will be deidentified, and the code will be retained by the Norwegian Patient Registry (NPR).

### Trial Design, Randomization, and Recruitment

**Figure 1.**
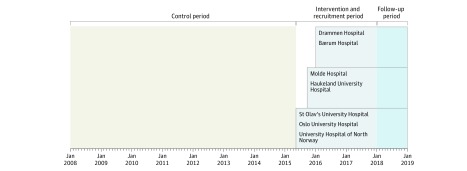
Norwegian Capture the Fracture Initiative (NoFRACT) Stepped Wedge Cluster Randomized Clinical Trial Design The 7 hospitals were randomized for the order of the starting dates and divided into 3 sequences. The intervention was introduced stepwise with 4-month intervals. The intervention period started on May 1, 2015, and will continue through December 31, 2018, with follow-up through December 31, 2019. The University Hospital of North Norway was scheduled to start on May 1, 2015, but was delayed for 5 months and started on October 1, 2015.

The hospitals were randomized to intervention starting date in a stepped wedge cluster randomized, open cohort design.^32,33^ The design was chosen because FLS has been shown to be of more benefit than harm, and this particular design is efficient for evaluating interventions that have previously been found to be effective in individually randomized studies. We sought to evaluate the effect of the intervention in a public health setting for implementation in clinical practice. In the design, the intervention is introduced to each cluster at regular time intervals. Each hospital acts as its own control, providing outcome data from before the intervention (2008-2015; control period) and after the intervention (2015-2019; intervention period). Randomization by lottery was conducted 8 weeks before the study start by an independent organization, the Norwegian Osteoporosis Association. The intervention was introduced with 4-month intervals between the clusters, and the 7 hospitals were divided into 3 sequences consisting of 2 to 3 clusters (hospitals) in each step. University Hospital of North Norway, St Olav’s University Hospital, and Oslo University Hospital were scheduled to start the intervention on May 1, 2015; Haukeland University Hospital and Molde Hospital on September 1, 2015; and Drammen Hospital and Bærum Hospital on January 1, 2016 (Figure 1). The recruitment phase has been ongoing from May 1, 2015, through December 31, 2018, with follow-up planned throughout December 31, 2019. Different patients were included at each step but subsequently followed up throughout the study period (open cohort design). The observation time in the intervention period (2015-2019) will range from 12 to 56 months for each of the patients in the study (unless an outcome or censoring takes place before the end of follow-up).

### Eligibility Criteria

Women and men 50 years and older with a recently diagnosed low-energy fracture who were admitted at 1 of the 7 hospitals were included, either while in the hospital or as outpatients within 6 weeks after the fracture. Patients with fractures of fingers, toes, skull, or face were ineligible. The very fragile patients, who were not expected to live long enough for the intervention to take effect as judged by the treating physician, will not receive the intervention. However, they will remain in the statistical analyses (intention to treat).

### Study Intervention

**Figure 2.**
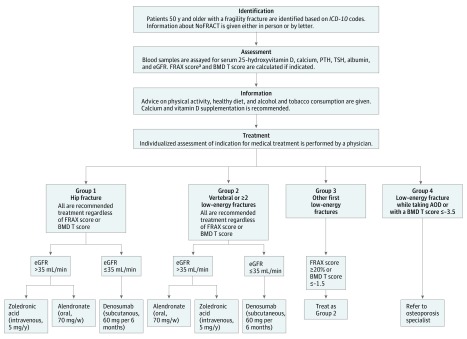
Application of the Standardized Intervention Program in the Norwegian Capture the Fracture Initiative (NoFRACT) Trial AOD indicates antiosteoporosis drugs; BMD, bone mineral density; eGFR, estimated glomerular filtration rate; *ICD-10*, *International Statistical Classification of Diseases and Related Health Problems, Tenth Revision; *PTH, parathyroid hormone; and TSH, thyroid-stimulating hormone. ^a^The Fracture Risk Assessment Tool (FRAX) is used to calculate the 10-year probability of major osteoporotic fracture (score is given as a percentage; a higher percentage indicates higher probability of fracture).

The intervention is a standardized program for identification, assessment, and treatment of osteoporosis in patients with a recently diagnosed low-energy fracture (a fracture occurring after a fall from standing height or less) and is based on the FLS model of care (Figure 2).

#### Identification

The coordinating nurse identifies the patients based on the hospital *International Statistical Classification of Diseases and Related Health Problems, Tenth Revision (ICD-10)* codes and eligibility criteria and provides information about the project either in person or in a letter sent to recently discharged inpatients and outpatients.

#### Assessment

Blood samples are obtained from the patients with fractures within 6 weeks of the index fracture to rule out common causes for secondary osteoporosis. The blood samples are assayed for serum levels of 25-hydroxyvitamin D, calcium, parathyroid hormone, thyroid-stimulating hormone, albumin, and creatinine. Kidney function is assessed using estimated glomerular filtration rate (eGFR).^34^ The BMD is measured using dual-energy x-ray absorptiometry of both hips and spine, and BMD T score is calculated. The 10-year probability of major osteoporotic fracture is calculated using the Fracture Risk Assessment Tool (FRAX; score is given as a percentage, with a higher percentage indicating a higher probability of fracture).^35^

#### Information

The coordinating nurse informs the patients on the importance of bone fragility and provides general lifestyle advice on physical activity, healthy diet, and tobacco and alcohol consumption according to national guidelines. All patients are recommended sufficient intake of calcium and vitamin D through diet or supplementation (500-1000 mg of calcium and 800 IU of vitamin D daily). Patients are also referred to fall prevention programs at the hospital or in primary care if the nurse finds this protocol to be relevant.

#### Treatment

Antiosteoporotic drugs are offered to 4 groups of patients. In group 1, patients with hip fracture are offered treatment regardless of BMD T score or FRAX score. The primary drug of choice for patients with hip fracture is (1) intravenous zoledronic acid (5 mg per year) to evade compliance problems, (2) oral alendronate (70 mg per week), or (3) subcutaneous denosumab (60 mg every 6 months).

In group 2, patients with vertebral fracture or 2 or more low-energy fractures are offered treatment regardless of BMD T score or FRAX score with (1) oral alendronate (70 mg per week), (2) intravenous zoledronic acid (5 mg per year), or (3) subcutaneous denosumab (60 mg every 6 months).

In group 3, patients with their first low-energy fracture are offered dual-energy x-ray absorptiometry for assessment of BMD T score, FRAX score, or both. The same treatment as in group 2 is offered to those with a BMD T score greater than −3.5 and less than or equal to −1.5 or FRAX score of major osteoporotic fracture of 20% or more.

In group 4, patients with a BMD T score of −3.5 or less or a subsequent low-energy fracture while taking anti-osteoporotic drug treatment for more than 1 year are referred to an osteoporosis specialist for consideration of bone anabolic treatment with teriparatide.

All patients are individually evaluated and treated according to comorbidities and kidney function. Bisphosphonates (alendronate or zoledronic acid) are the drugs of choice unless the patient had an eGFR of 35 mL/min or less; those patients are offered denosumab treatment unless they have an eGFR of 20 mL/min or less or any other contraindication was present. Patients with elevated serum levels of parathyroid hormone and calcium are referred to an endocrinologist, whereas patients with smaller deviations in those levels are followed up for 2 to 4 weeks after treatment initiation in primary care, as are patients with elevation of thyroid-stimulating hormone levels.

Length of treatment is dependent on the type of drug. For alendronate, a treatment break is recommended after 5 years; for zoledronic acid, a treatment break is recommended after 3 years; and for denosumab, no treatment break is recommended.

### Adherence to the Protocol and to the Intervention

The coordinating nurses at the NoFRACT hospitals underwent training in FLS with a well-trained nurse and physician to ensure standardization of the program. The University Hospital of North Norway was scheduled to start on May 1, 2015, but was delayed for 5 months and started on October 1, 2015. All other hospitals initiated the intervention on the date as scheduled.

The patients who were prescribed treatment with antiosteoporotic drugs were offered follow-up with the coordinating nurse. After 3 months, a phone consultation was performed to rule out misunderstandings, answer questions, and take care of potential adverse events. The close follow-up was conducted to improve the adherence to treatment. After 12 months, the patients were again offered a phone consultation or an appointment with a nurse. Each patient and their general practitioner were advised concerning length of treatment, and the patient could be referred for a reassessment after 2 to 3 years to decide further treatment.

### Potential Harm From the Intervention

Exposure to the intervention is associated with minimal discomfort; however, blood samples are obtained at baseline (start of the intervention), during which some patients may experience a slight discomfort. Dual-energy x-ray absorptiometry is a painless imaging modality with a modest dose of radiation.^36^ The most common adverse effects of antiosteoporotic drugs are muscle pains and gastrointestinal symptoms, the latter of which are caused by oral bisphosphonates.^37^ Intravenous treatment with zoledronic acid is associated with transient hypocalcemia and influenzalike symptoms. In addition, bisphosphonates are rarely associated with risk of osteonecrosis of the jaw and atypical femoral fracture.^37^ The patients were informed of potential adverse effects from the treatment.

### Study Outcomes

The primary study outcome is change in the rate of subsequent fracture (per 10 000 person-years) for patients with the following *ICD-10* codes: S22 (rib[s], sternum, and thoracic spine), S32 (lumbar spine, pelvis), S42 (shoulder, upper arm), S52 (forearm), S62 (wrist, hand), S72 (femur), S82 (patella, lower leg, and ankle), and S92 (foot). The secondary study outcome is all-cause mortality among patients with the following *ICD-10* codes: S72.0-S72.2 (hip fracture).

### Sample Size Determination

Initially, the plan was to include 87 000 patients based on sample size calculations performed before starting the trial (during 2008-2017) (ie, approximately 9600 patients each year). Local counting of patients exposed to the NoFRACT intervention at the 7 Norwegian hospitals during 2015-2017 showed that approximately 7500 patients with fracture had been included annually. Because the recruitment of patients had been somewhat lower than expected, a decision was made to continue the recruitment for 1 additional year throughout 2018, with data obtained from registers throughout 2019 (to achieve at least 1 year of observation time for all patients). There are 4 months between the steps in our design (3 steps per year) at the 7 hospitals; this design gives a mean cluster size of 357 patients (7500/[7 × 3] = 357). Because the baseline data from patients will be used for a total of 11 calendar years (2008-2018), a revised estimate of the number of patients who will be included is 82 467 (357 × 7 × 3 × 11 = 82 467; approximately 56 000 patients in the control period and 26 000 in the intervention period). Power calculations using the “steppedwedge” command in Stata, version 15 (StataCorp), show that we have 80% power to detect a relative risk of 0.73 for any type of subsequent fracture in the intervention period vs control period. This figure assumes inclusion of 82 467 patients with fracture (any fracture type), intracluster correlation of 0.03, and cluster size of 357. The intracluster correlation was calculated as in a previous study.^3^ In these calculations, the proportions of subsequent fracture in the intervention and control periods were estimated to be 6% and 9%, respectively, after 1.5 years based on previous FLS studies.^13,14,17^ Estimates from the Swedish study by Axelsson et al^13^ (after 0.9 years of follow-up: 6.6% in the treated group vs 8.8% in the untreated group; relative risk, 0.75) were given the most weight because Sweden, similar to Norway, has high fracture rates^24^; however, the incidence of fractures estimated in other relevant studies were also considered.^3,27,38,39^

Regarding hip fracture risk among patients with a first fracture of any type, power calculations give a relative risk of 0.52 in the intervention vs control periods, assuming 80% power, inclusion of 82 467 patients with fractures, proportions of patients with a subsequent hip fracture of 3.0% in the control period and 1.6% in the intervention period, an intracluster correlation of 0.03, and a cluster size of 357.^3^

### Data Sources and Collection

Outcome, time at risk, and potential confounding factors have been obtained since January 1, 2008, and will continue to be collected through December 31, 2019, from the following national registries: the NPR, National Population Register, Statistics Norway, and Norway Control and Reimbursement of Healthcare Claims (KUHR) (Table 2). The NPR provides data on patients with fractures treated at Norwegian hospitals. Fracture diagnoses other than hip fracture (which is always treated in hospitals) is also complemented by the KUHR database, which comprises fractures treated by primary care physicians and emergency units in rural and semirural areas.

**Table 2.** National Registries Used for Outcome Assessment in the Norwegian Capture the Fracture Initiative (NoFRACT) Study

Registry	Variable	Type of Fractures
Norwegian Patient Registry	Sex, birth year, hospital, hospitalization dates, municipality of residence, treatment level, surgical procedure codes, and Charlson comorbidity index	Fractures treated in hospitals
National Population Register	Dates of migration and death, marital status, and country of birth	All fractures
Statistics Norway	Education level	All fractures
Norway Control and Reimbursement of Healthcare Claims	*ICPC-2* diagnosis codes L72-L76, including subgroup	Fractures treated in primary care

Abbreviation: *ICCP-2, International Classification of Primary Care, 2nd ed.*

### Validity

The use of registry data are highly dependent on the quality of the registries. The accuracy of hip fracture diagnosis in the NPR has been evaluated and found to be 93.5%.^40^ A study of the distribution and degree of overlap of fracture diagnoses in the NPR and KUHR will be performed. Ahead of our main analysis, the accuracy of the diagnosis of forearm fracture will also be evaluated in a separate validation study that includes fractures from hospitals (registered in the NPR) and from the primary health care service (registered in the KUHR).

### Data Management

All data will be deidentified by replacing the personal identification number with a project-specific identification number for each individual. The code (personal identification vs project-specific identification) will be retained by the NPR. The data will be securely stored at a protected platform for research at the University of Oslo, Services for Sensitive Data, which meets all Norwegian law requirements. Data will be available only to collaborators with the approval by the Regional Committee for Medical and Health Research Ethics, Region South Eastern Norway. All data management (eg, quality control and linking of data sources) will be performed within the Services for Sensitive Data.

### Data Analysis Plan

Outcome data from the control period and intervention period will be compared. The date of the index fracture (first fracture in the trial period) determines whether a patient’s data will be included in the control or intervention period. The study will use time-to-event analysis, in which time at risk of subsequent fractures will be calculated based on first fracture dates (hospitalization dates or primary health care treatment dates), dates of migration or event occurrence (death from the National Population Register or subsequent fracture from the NPR), or the end of the study (December 31, 2019). Using calendar time as the scale will allow observation of secular changes in subsequent fracture risk over time, and if necessary, differences in secular trends between hospitals will be considered by including an interaction term between cluster and time. Clustering by hospital will be included as random effects in mixed models to estimate incidence rate ratios with 95% CIs. A uniform correlation structure is assumed,^41^ with no decay in the cluster autocorrelation. All patients treated for relevant fractures and residing in a municipality belonging to 1 of the 7 hospitals after initiation of the intervention will be allocated to the intervention group irrespective of exposure to the intervention (intention to treat). Because risk of subsequent fracture varies by follow-up time and fracture type,^3,39^ additional analyses will be performed in which the same maximum follow-up time (1 year) after the index fracture will be used for patients in the control period.

The need for a transition period (period for the intervention to be established) to minimize within-cluster contamination will be explored in sensitivity analyses. The pattern of missing data will be examined, and if necessary, multiple imputation will be performed based on the collected exposure data, covariates, outcome, and intracluster correlation. Analyses will be stratified by sex, and subsequent fracture rates and mortality rates in women and men will be compared. The analyses will be adjusted for potential confounders, such as age, education, and comorbidity (Charlson index score).

### Trial Status

At the beginning of January 2018, there were 23 578 patients enrolled in the intervention period of the NoFRACT study of the approximately 26 000 patients planned.

## Patient and Public Involvement

The development of NoFRACT was motivated by previous studies that showed undertreatment of osteoporosis in patients with fragility fractures.^7,8,9,10^ Patients were not involved in the initial development of the study design, conduct, and recruitment. However, 2 patients with a previous fracture who have been receiving antiosteoporotic drugs have been involved in the later stages of the study and contributed to the development of new research questions. The patients were recruited through the NoFRACT network and were interviewed regarding preferences and experiences; they also commented on the trial protocol. Patients’ priorities, experience, and preferences have also been discussed in public meetings.

## Dissemination Plan

The dissemination plan includes publication of positive, negative, and inconclusive results in peer-reviewed Norwegian and international scientific journals. Primary and secondary outcomes will be presented in separate papers. Authorship for the scientific papers will be settled according to the Vancouver protocol and the International Committee of Medical Journal Editors recommendations. The results from the study will be presented at national and international academic meetings. The protocol and journal publications will be made available to the public; however, because of strict protection of privacy under Norwegian law, individual-level data sets can only be shared if approved by the Regional Committee for Medical and Health Research Ethics.

The results from the study will be disseminated to the public through newspapers, television, public meetings, and social media in collaboration with 2 patient advisors. To reach the patients more directly, the results will be communicated to treating physicians and hospital staff and through patient organization. Implementation of research results into clinical practice is important. It is therefore necessary to communicate research results and clinical treatment strategies to decision makers to ensure that best clinical practice is implemented and made equally available for the patients in Norway and worldwide.

## Conclusions

By introducing a standardized intervention program for assessment and treatment of osteoporosis in patients with fragility fractures, we expect to document reduced rates of subsequent fractures and fracture-related mortality.

## Notes

Supplement 1. **Trial Protocol**

Supplement 2. **Data Sharing Statement**
