# Supplementary material for: Effect of a Fracture Liaison Service on the Rate of Subsequent Fracture Among Patients With a Fragility Fracture in the Norwegian Capture the Fracture Initiative (NoFRACT): A Trial Protocol
Source: JAMA Netw Open. 2018 Dec 7;1(8):e185701. doi: 10.1001/jamanetworkopen.2018.5701 (PMC6324344; doi:10.1001/jamanetworkopen.2018.5701)
Supplement: Supplement 1. — Trial Protocol [file jamanetwopen-1-e185701-s001.pdf]

NoFRACT - Norwegian Capture the Fracture Initiative

1. Relevance relative to the call for proposals

Bone fragility is a major public health problem due to the accompanying increased morbidity, mortality and financial cost as a result of fractures. The increase in life expectancy of the population means that the annual number of fractures and associated costs are expected to increase by 50% between 2005 and 2050. Despite the high economic cost to society and personal cost to affected individuals, fracture prevention in Norway is suboptimal, as many patients do not receive available and efficient therapy. A prior fragility fracture almost doubles a patient's risk for a future fracture and multiple fractures increase the risk up to five fold. Still, the majority of fragility fracture patients are neither assessed, nor treated for osteoporosis. In Norway, as in many other countries there is a large care gap leaving millions of fracture patients at serious risk of future fractures. A fracture liaison service model of care is recommended, but data on its effectiveness is scarce. "Norwegian Capture the Fracture Initiative", is a cross-regional cooperative project based on initiatives of several hospital departments and universities, which aims at closing this gap by improving secondary fracture prevention. By introducing a standardized intervention program for assessment and treatment of fracture patients, we expect to document reduced rates of fractures and fracture related mortality. Seven Norwegian hospitals are randomized for the starting date of the study in a Stepped Wedge Cluster Randomized Controlled Trial design. The effect of the intervention will be measured based on endpoints from national registers. Each hospital will act as their own control, and endpoints will be compared before and after the intervention.

2. Aspects relating to the research project

The main aim is to assess the effectiveness of introducing a standardized intervention program for treatment of fragility fracture patients, on the fracture rates and mortality across regions of Norway.

2.1. Background and status of knowledge

Epidemiology of fragility fractures

Bone fragility is a global health problem, and for largely unknown reasons Norway has the highest rates of fractures in the world.[1;2] As the population ages, the annual number of fractures and associated costs are expected to increase by 50% between 2005 and 2050.[3;4] If hip fracture rates in

Norway remain constant the burden is expected to double towards 2040.[5] Women and men above 50 years of age have a remaining lifetime risk of fractures of 46% and 22%, respectively.[6] Fragility fractures are associated with a substantial burden of morbidity as pain, loss of function, disability, hospitalization, and long-term nursing care.[7;8] In Europe, about 2.7 million fragility fractures occur every year in men and women, and the direct annual cost of this is estimated to EUR 36 billion.[9] Despite the high economic cost to society and personal cost to affected individuals, osteoporosis prevention is suboptimal, as many patients do not receive available and efficient therapy.[3] The anti-osteoporosis drugs are readily available and may reduce the risk for future fracture by 30-50%.[10;11]

In Norway, about 10 000 subjects over 50 years of age suffer a hip fracture every year, [12;13] and the annual number of forearm fractures is estimated to 15 000.[14;15] As hip fractures are estimated to constituted 20% of all osteoporotic fractures in Europe,[16] this would imply 50 000 osteoporotic fractures annually in Norway, but exact number is lacking. Moreover, there is no good estimate of the total costs of osteoporotic fractures in Norway. Folkehelsemeldingen 2012/2013 (report from the Norwegian government) refers to hip fractures as one of the most expensive diagnoses for the Norwegian health system, and the total costs during the first year after a hip fracture is estimated to NOK 500.000.[17] In Sweden, the annual costs related to fractures were estimated to SEK 5.6 billion.[18] Acute treatment in hospital accounted for 1/3 and public services as nursing homes accounted for 2/3 of the total costs. Including quality-adjusted life-years (QALYs) lost; the annual societal burden of fragility fractures in Sweden of SEK 15.2 billion in 2005 is expected to increase by 56 % to 26.3 billion in 2050.[18]

The pathogenesis and clinical significance of bone fragility

The pathogenesis of fractures is multifactorial; genetics, trauma mechanism, falls and bone strength are all important factors.[19] Women suffer more fractures than men, particularly postmenopausal women, but the risk of recurrent hip fracture is similar given similar remaining life expectancy.[13] However, compared to women, men have higher mortality after a hip fracture,[20] and the 1-year excess mortality post hip fracture was 2.8 and 4.6 increased in women and men, respectively.[21] One year after a hip fracture, one of four patients over 50 years of age were no longer alive.

Assessment and treatment of persons at high risk of fracture

Measurement of femoral neck bone mineral density (BMD) by dual energy X-ray absorptiometry (DXA) is the most common approach used to assess fracture risk and is considered the gold standard surrogate for bone strength.[22;23] However, although a lower BMD is associated with increased risk of fracture, most people with fractures do not have osteoporosis (T score of 2.5 or

more standard deviations (SD) below the young normal mean) but osteopenia or normal BMD.[23;24] To address this lack of sensitivity, Fracture Risk Assessment Tool (FRAX) is developed, which calculates the 10-years probability of a major osteoporotic fracture based on clinical risk factors as age, height, weight, smoking, excessive alcohol intake, previous fracture, parental history of hip fracture, glucocorticoid therapy, rheumatoid arthritis and femoral neck BMD.[25-27] Whereas Garvan nomograms are based on age, BMD, prior fracture and prior falls.[28] These tools are easy available online. A prior fragility fracture almost doubles a patient's future fracture risk and multiple fractures increase the risk up to five fold.[29] About 75% of re-fractures occur within five years after a first hip fracture.[30] Unfortunately, in Norway, few patients have a DXA scan done; only 14.6% of women and 4.2% of men receive anti-osteoporosis drugs after a hip fracture,[31] and only 20% and 35% of patients with osteoporosis aged 60-69 and 70-79 years, respectively.[32]

Fracture liaison services

A fracture liaison service (FLS) model of care is a systematic approach to secondary fracture prevention, including a dedicated coordinating nurse.[33] After Glasgow University Teaching Hospitals introduced FLS in 1999, the hip fracture rate was reduced by 7.3%, while at the same time, the rate increased by 17% in England. Per 1000 patients assessed by the FLS, 18 new fractures were prevented, which included 11 hip fractures.[34] Likewise, Kaiser Permanente introduced The Healthy Bone Program in Southern California, USA in 2001. In 2006 actuarial analyses expected 2510 hip fractures, but only 1575 hip fractures were observed, indicating a decrease in hip fracture rate by 37%.[35] This led to a cost-reduction of USD 30.8 million in 2006.[35] The Academic Hospital of Maastricht employed a coordinating nurse in 2005, which increased the number of DXA scans compared with the surrounding hospitals.[36] The Concord Repatriation General Hospital, Sydney established their Minimal Trauma Fracture Liaison Service in 2005. Patients involved in the program had 80% lower incidence of new fractures than controls.[37] At Skåne University Hospital in Lund, Sweden the risk of new fractures was reduced by 42% after introduction of osteoporosis assessment of patients with fragility fractures (wrist, shoulder, vertebral, or hip fracture), and mortality after fractures was slightly reduced.[38;39] However, most of these studies are relatively small, and robust data on the effectiveness of FLS is scarce. Thus, larger randomized controlled trials with fracture risk and mortality as primary end-points are needed.

There are no systematic routine or national guidelines in Norway pertaining to FLS, most patients with a fracture are not offered assessment and secondary fracture prevention. This may be due to lack of knowledge or interest for this topic among health professionals. Compliance and

resilience are additional challenges. This failure gives, however, an opportunity for a large-scale evaluation of the effect and cost effectiveness of the FLS concept, which is very much in demand.

2.2. Approaches, hypotheses and choice of method

To solve these issues, initiatives are taken by several orthopedic surgeons, endocrinologists, rheumatologists and scientists at seven small and large hospital departments and universities from all four health regions in south-east, west, central and north of Norway, to collaborate on this patient-oriented clinical research project. The population and total number of hip, wrist and proximal humerus fractures in patients ≥50 years at each of each hospital in 2013 are shown below.

	**Oslo**[a]	**Bærum**	**Drammen**	**Bergen**[b]	**Molde**	**St Olav**	**Tromsø**
Population (in 1000)	**500**	**180**	**140**	**500**	**70**	**300**	**120**
Hip	600	350	261	450	123	402	180
Forearm/wrist	950	550	414	1000	168	397	347
Proximal humerus	590	200	160	600	68	197	88

[a]Oslo University Hospital including “Legevakta”, [b]Haukeland Hospital including “Legevakta”

This cross-regional cooperative project is a unique opportunity to measure the effect of introducing a standardized intervention program, mainly in terms of possible changes in fracture rates and fracture-associated excess mortality. The effect of this intervention will be measured based on endpoints from national registers (as outlined below).

The main aim is to assess the effectiveness of introducing a standardized intervention program for treatment of fragility fracture patients measured by changes in recurrent fracture rates and mortality.

A fracture liaison service (FLS) model of care is widely recommended but data on its effectiveness regarding recurrent fracture risk and fracture related mortality is scarce. We therefore aim to assess the effectiveness of an intervention in terms of introducing a standardized program for assessment and treatment of bone fragility in fracture patients. This FLS program will involve dedicated coordinating nurses at each hospital, who will approach fracture patients, invite them to participate, inform them about the importance of bone fragility, give lifestyle advice concerning physical activity, healthy diet, moderate alcohol intake and smoking cessation, and offer assessment and treatment for osteoporosis if needed. Seven hospitals are Stepped Wedge Cluster Randomized for

starting date of introducing a standardized intervention program from 2015 to 2016, with follow-up throughout 2019 (Fig. 1). The effect of the intervention will be measured based on endpoints from national registers. Main outcomes are recurrent fractures (hip, forearm and all fracture types) and post hip fracture mortality. Each hospital will act as its own controls, and provide endpoint data before and after intervention. In the analyses the effect of the standardized intervention program will be estimated by comparing data from before (2008-2014), and after the intervention (2015-2018).

We believe this project will generate new knowledge by providing evidence on how to improve patient care and reduce the accelerating fracture-related health care costs on health budgets by prevention of secondary fracture, reduce morbidity, mortality and improve quality of life. Moreover, this will enhance the health service’s competence and quality and contribute to the development of new national guidelines and the health services delivered by hospitals. Interdisciplinary programs and clinical pathways to improve the care of patient groups have increased within the health services the past 20 years, and large scale evaluations of clinical effects and costs are needed.

Testable Research Questions and Hypotheses

1. Incidence of forearm fractures and other low-energy fragility fractures at different hospitals and regions of Norway will be assessed in register data before intervention.
2. The incidence of fragility fractures (hip, proximal humerus and wrist) will be reduced at hospitals with a standardized intervention program compared to hospitals without.
3. The mortality rate after fragility fractures (hip, proximal humerus and wrist) will be reduced at hospitals with a standardized program compared to hospitals without.

Stepped Wedge Cluster Randomized Controlled Trial

In this stepped wedge cluster randomized controlled trial, the hospitals are randomized for the order to start of the study. The intervention was planned to start at 4 months interval for 3 clusters (consisting of 2-3 hospitals, Fig. 1), until all 7 hospitals have received the intervention. Randomization was conducted 8 weeks prior to the start of the study, by the leaders of the Norwegian Osteoporosis Association, who are not involved as collaborators in the study:

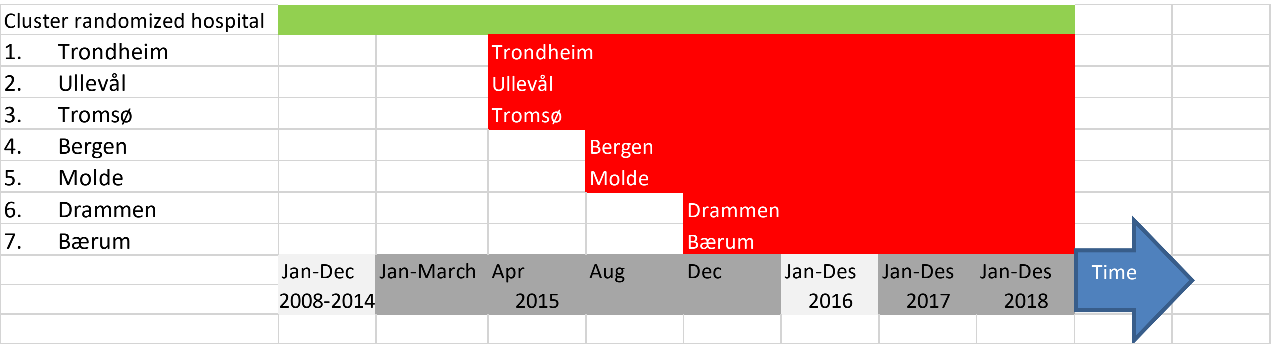

Fig. 1. Study design - stepped wedge cluster randomized controlled trial

April 2015, St. Olavs hospital, Oslo University Hospital, University Hospital of North Norway, Aug 2015: Haukeland University Hospital and Molde Hospital, December 2015: Drammen Hospital and Bærum Hospital. However, this plan has been delayed by one month so starting time points for each cluster was: May 2015, September 2015, and January 2016. St. Olavs hospital and Oslo University Hospital started May 2015, Haukeland University Hospital and Molde Hospital started September 2015, the University Hospital of North Norway started October 2015. Drammen Hospital and Bærum Hospital are preparing to start January 2016. The recruitment phase of 3-3.5 years will last from the beginning of the study until end of December 2018, with the follow-up after one year, during 3-3.5 years from May 2016-December 2019.

A stepped wedge design is similar to a crossover design in terms that the different clusters (groups of hospitals) in turn switch from no standardized program (control) to standardized program but at different time points. The clusters will cross over in only one direction.[40;41] Each hospital will be their own control, and endpoints will be compared before and after the introduction of intervention. This stepped wedge cluster randomization design is particular relevant where it would be considered unethical to not deliver or retract the intervention when it is expected to do more good than harm, as here where the intervention is expected to be included as a future standard routine.

Standardized Intervention Program initiated from May 2015 (see Fig. 2 below)

1. Women and men ≥ 50 years of age with a low-trauma vertebral or non-vertebral fracture (except fingers, toes, face and skull) will be approached by the coordinating nurse, and offered information, assessment, lifestyle advice and treatment for bone fragility, preferable while in-patient are at the hospital, or as out-patients, within 6 weeks after the fracture (see algorithm Fig. 2). Lifestyle advice about physical activity, fall prevention, healthy diet, smoking cessation and moderate alcohol intake, will be given, and referral to fall prevention at the hospital or primary care if indicated.

Exclusion criteria will be age below 50 years and short life expectancy. The project nurse in collaboration with the physician will perform individual assessment of eligibility.

**Inclusion criteria**	**Exclusion criteria**
≥ 50 years and low-trauma fracture*	<50 years
	Fractures of skull, face, toes, or fingers
	Short life expectancy
	Patient refuses assessment or treatment

*low-trauma fracture will be the key target group, however, sometimes it is difficult to tell whether high-trauma or low-trauma is involved in the fracture event, thus high-trauma may also be included

2. Blood samples will be collected and serum assayed for 25-hydroxyvitamin D (25[OH]D) (using mass spectrometry, LCMS-MS (Oslo, Drammen, Bergen and Tromsø), or using immunoassays (Bærum, Molde and Trondheim)), calcium, parathyroid hormone (PTH) (Immulite 2000), kreatinine (for estimation of glomerulous filtration rate (eGFR)) and thyroid-stimulating hormone (TSH). These measurements are needed for individual treatment decision as shown below.

3. Bisphosphonates are contra-indicated if kidney function is reduced (eGFR ≤ 35 ml/min), and drug of choice is anti-RANKL, if eGRF is ≤ 35 ml/min and > 20 ml/min. All fracture patients with reduced kidney function (eGFR<35 ml/min), will be considered for treatment with Denosumab except if eGFR <20 ml/min or any of the exclusion criteria are present. Regular monitoring of S-Ca is required when treating patients with reduced kidney function. Whether patients with severe kidney failure (dialytic or predialytic stage) or kidney transplantation can be treated with Denosunab, but require careful consideration and individual assessment by a kidney specialist in collaboration with an expert on osteoporosis treatment (e.g. endocrinologist or rheumatologist).

4. Patients with low serum levels of calcium will be treated with calcium and Vitamin D supplementation for 2 weeks before anti-osteoporosis drug (AOD) treatment will be initiated.

5. Patients with elevated S-Ca and PTH > 15 will be referred to endocrinologist. Smaller deviations in s-Ca and PTH will be followed by fasting blood samples after 2-4 weeks. Patients with significant deviation in TSH will be referred to their GP for further investigation of thyroid function.

6. Basic recommendation for supplementation is: 800 IU (20µg) vitamin D + 500-1000 mg calcium.

7. Hip fracture patients often have low levels of Vitamin D, and they will be recommended treatment with an initial dose of 100 000 IU Vitamin D x1 p.o. or i.im if their kidney functions is not reduced (eGRF > 35 ml/min). If they have reduced kidney function (eGRF ≤ 35 ml/min) a nephrologist will be consulted for the dosage of active vitamin D (kalsitriol, 1-α,25-dihydroksy vitamin D_3), calcium supplement and if there are any contraindications to AOD (high risk of renal osteodystrofia e.g.).

8. BMD will be measured of both hip and spine, with morphometric assessment of vertebral fracture assessment on lateral x-ray (VFA) (Lunar Prodigy DXA, Madison, WI, USA) if possible. All will be offered a DXA scan at hospitals with DXA available (for BMD T-score or FRAX score calculation) except patients with dementia, difficulties laying on the back, or short life expectancy; they will not be scanned by DXA (but have treatment decision made based on clinical assessment and FRAX score calculated without BMD). At hospitals without DXA machine available, FRAX score will be calculated for all fracture patients without BMD measurements.

9. Patients with fracture of the hip, vertebrae or 2 or more low-trauma fracture do not need DXA for treatment decision, however, a DXA scan for T-score or FRAX score calculation is needed for the decision about AOD treatment of patients with one other low-trauma fracture.

10. Hip fracture patients will be recommended AOD regardless of FRAX or T-score, and they do not need any DXA scan or FRAX score for treatment decision. Drug of choice i) zoledronic acid 5 mg iv x 1/year, to avoid compliance problem, ii) denosumab 60 mg sc/6 month to women > 75 years of age if they are not able to use peroral treatment, or iii) alendronate 70 mg po x 1/week.

11. Patients with vertebral fracture or ≥ 2 low-trauma fracture will similarly be recommended AOD treatment regardless of T-score or FRAX score, as outlined in the treatment algorithm below.
Drug of choice i) alendronate 70 mg po x 1/week, ii) zoledronic acid 5 mg iv x 1/year, or iii) denosumab 60 mg sc/6 month.

12. For patients with one other low-trauma fracture; a DXA scan is recommended, and AOD treatment is recommended if T-score ≤ -1.5 or FRAX score for major fracture ≥ 20%. If kidney function is normal, the first choice of AOD is alendronate 70 po/week, see treatment algorithm.

13. Patients with spine or femoral neck T-scores ≤ -3.5, > 2 severe vertebral fractures (> 40% compression), and those who suffer a second fracture while using AOD, will be referred for further examination by specialist at the hospital and teriparatide treatment will be considered.

14. AOD will be prescribed by a hospital physician involved in this project. All patients treated with AOD will be offered a follow-up phone call after 3 months by the nurse and a visit to talk with the nurse after 1 year, to improve the compliance and resilience.

In brief, at all hospitals, the intervention is a standardized program involving a nurse and physician who offer information, assessment, and treatment if needed in the hospital setting as an integrated part of the fracture treatment. All patients included in this study, will be offered a follow-up appointment to talk with the nurse 1 year after the fracture. The study design is kept simple to make the project realistic and feasible, scientifically, organizationally, in relation to the use of resources.

Treatment Program for women and men ≥ 50 years after low-trauma fracture

NoFRACT Norwegian Capture the Fracture® Initiative

All hospital pasients will be offered*

- Optimal fracture treatment
- Assessment of bone fragility using DXA scan and FRAX score with follow-up appointment
- Treatment for bone fragility – lifestyle advice and anti-osteoporotic drug as outlined in this program
- Blood samples
- Fall prevention, e.g. consider referal to pysio- or ergotherapist and/or fall out-patient clinic

*All patients will be approached after a vertebral or any non-vertebral fracture (except fractures of fingers, toes, face and skull).
Patients with dementia, difficulties laying on the back, or short life expectancy will not be offered DXA and FRAX score can be assessed without BMD. At hospitals without DXA, FRAX score can be assessed without BMD
(FRAX available: *http://www.shef.ac.uk/FRAX/tool.aspx?country=42)*

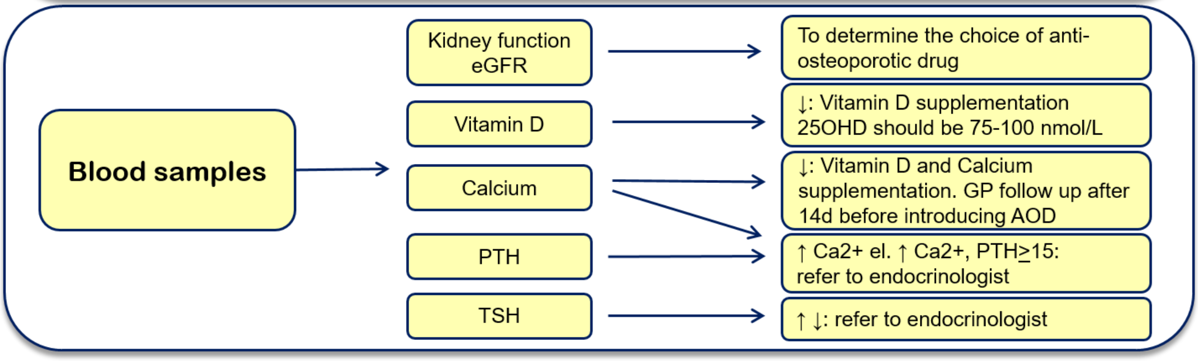

All fracture pasients will be offered DXA, lifestyle advices and recommended Vitamin D and Calcium suppl.*

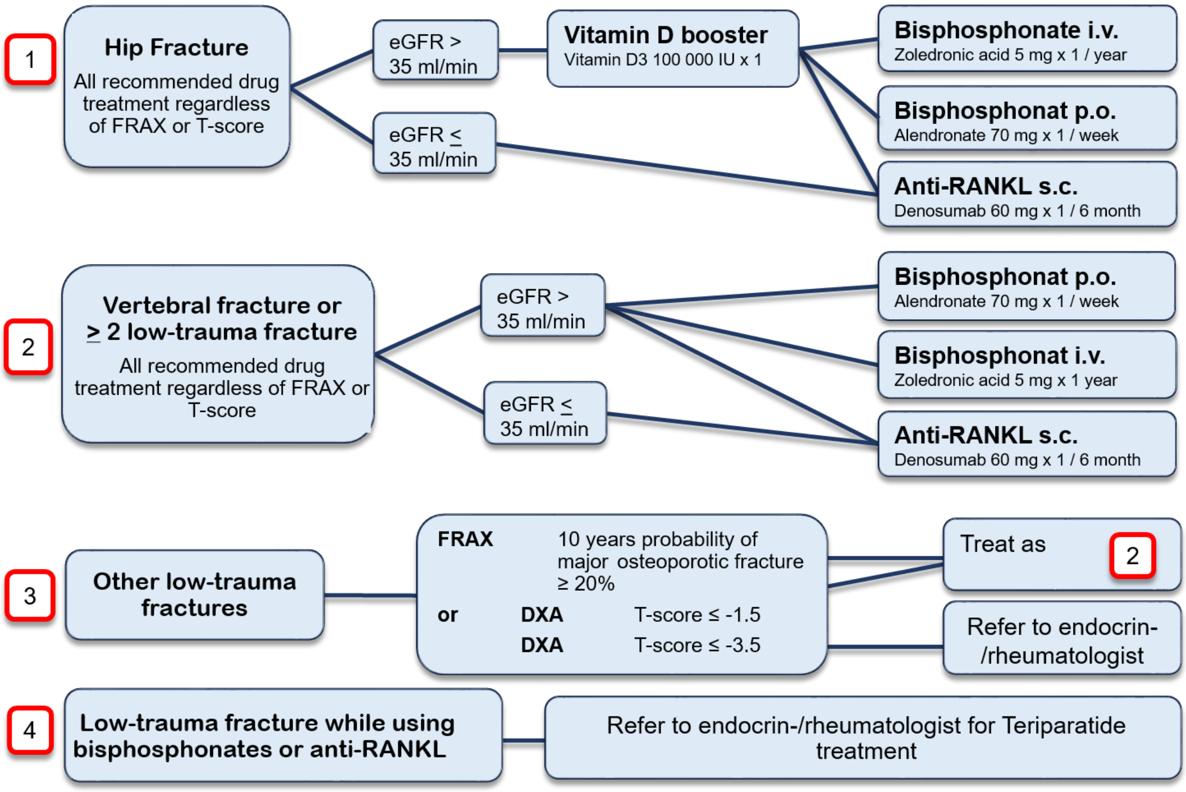

*Patients with dementia, difficulties laying on the back, or short life expectancy will not be offered DXA and FRAX score can be assessed without BMD. At hospitals without DXA, FRAX score can be assessed without BMD (FRAX available: *http://www.shef.ac.uk/FRAX/tool.aspx?country=42)*

Standardized Intervention Program was initiated from May 2015 (Fig. 2 above)

<u>Data from nationwide registers</u>

Data from national registries will be collected from 1 January 2008 to 31 December 2019.

Norwegian Patient Register: Data on fractures 2008-2019 treated in Norwegian hospitals will be retrieved from the National Patient Registry (NPR) *Charlson comorbidity score* –will be calculated based on all available diagnosis codes before a registered event. All hospital admissions after fracture will be retrieved, to evaluate total costs for all patients during follow-up.

Population register: Dates of death and migration, marital status and country of birth for all fracture patients will be obtained from the National Population Registry 2008-2019.

Statistics Norway: Information on education level in four categories is registered on the entire population of Norway and will be used as proxy for social class.

Control and reimbursement of healthcare claims (KUHR): Hip fractures are always treated in hospitals. However, patients with other types of fractures might seek private clinics, primary physicians or private x-ray institutes when they fracture. This type of information is available in KUHR. In order to make sure that information on fractures treated outside hospitals is not missed, we want to include KUHR-data. The ICPC-2 diagnosis codes L72-L76 contain relevant information on fractures (Fig. 3).

Merging and storing of data: Data from the Norwegian Patient Register (NPR), the National Population Registry (from 1 January 2008 to 31 December 2019), and statistics Norway will be combined (Fig. 3). Regarding fractures of the hip, additional information will also be obtained from the Cause of Death Register. The University of Oslo (UiO) has implemented a research platform Services for Sensitive Data (TSD), which meets all requirements in the Norwegian law regarding safe handling and storing of sensitive data. All data will be stored in TSD.

Statistics

In this stepped-wedge CRT, each of the hospitals will act as their own controls, and provide data from before and after intervention. In the analyses the effect of introducing the standardized intervention program will be estimated by comparing data before (2008-2014), and after the intervention (2015-2018), using stepped wedge CRT methods in STATA. Main outcomes are recurrent fractures (hip, forearm and all fracture types) and post hip-fracture mortality.

Power calculations including the seven hospitals show that we are able to find a relative risk (RR) of 0.56 for recurrent hip fracture risk in hip fracture patients (assumes inclusion of 56,000 person

years 2008-2017, 80% power, 95% confidence intervals, cluster coefficient of variation = 0.3) after intervention compared to before. Corresponding power calculations for any recurrent fracture in all fracture patients show we can find a RR of 0.76 after intervention compared to before (assumes inclusion of 260,000 person years).

We will study incidence of forearm fractures and other low-energy fragility fractures in registers prior to the intervention. We can also investigate to what extent there have been different time trends in fracture rates in hospitals included in the intervention versus those not included. The analyses will be adjusted for individual Charlson comorbidity score, and marital status.

Register	Variable	Concerning type of fractures
**Norwegian Patient Register**	Gender, birth year, hospital, hospitalization dates, municipality of residence, treatment level, surgical procedure codes and Charlson comorbidity index	Fractures treated in hospitals
**National Popualtion Register**	Dates of migration and death, marital status, country of birth	All fractures
**Statistics Norway**	Education levels	All fractures
**Control and Reimbursement of Healthcare Claims (KUHR)**	ICPC-2 diagnosis codes L72-76 including sub-groups	Fractures treated in primary care

Abbreviations: ICPC-2: International Classification of Primary Care 2 edition

Fig. 3. The national registers used for outcome assessment in the NoFRACT study.

2.3 The project plan, project management, organization and cooperation

The 5 years cross-regional project period will last from May 1, 2015 to April 31, 2020.

2015-2018 Coordinating nurses will recruit fracture patients at all seven hospitals, Oslo University Hospital (Ullevål), Bærum Hospital, Drammen Hospital, Haukeland University Hospital (Bergen), Molde Hospital, St. Olavs University Hospital (Trondheim) and University Hospital of North Norway (UNN, Tromsø). All fracture patients will be offered assessment and treatment as outlined previously. The dedicated FLS nurses will perform weekly systematic searches of all in-patients and out-patients, and approach admitted patients and invite out-patients to participate by letter, SMS

and telephone and offer appointment with nurse within 6 weeks. Good routines will be established together with our international collaborators, who have experience from establishing and maintaining such projects. The nurses will provide patients with information about bone fragility, and the impact of their future fracture risk, so they will be well motivated for modifying amendable lifestyle factors such as; physical activity, healthy diet, with sufficient intake of vitamin D and calcium, protein, avoid excess alcohol intake and quit smoking. Follow-up appointment with the nurses after 1 year will be offered for assessment of compliance. A physician will prescribe anti-osteoporosis drug (AOD) for those who need it and outlined in the treatment algorithm.

2018-2019 Data from registers will be retrieved for the first publications on the incidence of forearm fractures and other low-energy fragility fractures at different hospitals and regions in Norway. The PhD student will take the courses they need for the PhD program.

2020 The final papers on the effect of the introduction of the standardized program will be written by the PhD student and researchers. In 2021 the PhD student will defend their theses.

Research Team

The research team has all the qualifications and resources needed to complete the study including equipment, methods and infrastructure, regional, national and international collaborators. Treatment of fracture patients will be coordinated across specialities, as the team includes orthopedic surgeons, endocrinologist, epidemiologists and rheumatologists with longstanding experience in treatment of patients with fractures and/or osteoporosis; some are world capacities in the field. This collaboration will promote national and international network building. The international cooperation will ensure that the intervention will be well planned, organized and executed. Initiatives are taken at seven hospitals, particular from the Norwegian Orthopedic Surgeon Society of Osteoporosis and Bone Health (FOB), and four universities with a range of experts involved:

Oslo University Hospital, Prof. Lars Nordsletten, orthopedic surgeon (Ullevål), Frede Frihagen, PhD, orthopedic surgeon, Prof. Erik Fink Eriksen, endocrinologist, Ruth Aga, MD, Ida Lund, MD, (Legevakten), Ass Prof. Torbjørn Wisløff, statistician and health economist, Anne Kristine Brekke, Sissel Knuts, Elise Berg Vesterhus, Janne Blegen Høglund, Ingvild Hestnes, Herdis Palmadottir, Malte Smidt, Janicke Rudie, Karine R Haugvad, Ellen Johansson, Mette Bentdal Larsen, nurses.

University of Oslo, Tone K Omsland, PhD, epidemiologist, Cecilie Dahl, PhD, postdoctor.

Bærum Hospital, Wender Figved, PhD, orthopedic surgeon, Ellen Tverå Langslet, orthopedic surgeon, Katrine A Askevold, Hildegunn Berger, Merete Finjar, nurse.

Drammen Hospital, Tove Tveitan Borgen, rheumatologist, key contact person, Lars Michael Hubschle, orthopedic surgeon, Hanne Louise Hoelstad and May-Britt Stenbro are nurses.

Bergen, Haukeland University Hospital, Jan-Erik Gjertsen, PhD, orthopedic surgeon, Ellen Apalset, rheumatologist, PhD, 2 nurses will be recruited as coordinators.

Molde Hospital, Lene B Solberg, PhD, orthopedic resident, Jens Stutzer, orthopedic surgeon, Charlotte Råmkes and Solveig Solberg, nurses.

Trondheim, St. Olavs University Hospital, Trude Basso, PhD, orthopedic resident, Lars Gunnar Johnsen, PhD, orthopaedic surgeon, Prof Unni Syversen, endocrinologist, Mari Hoff, PhD, rheumatologist, Sølvi Liabakk, physiotherapist, Nina Raaness Larsen, Kristine Aavik Haugen, Gry Mette Torstensen and Hilde Kjøsnes Thoresen, nurses.

Norwegian University of Science and Technology, Gunhild Hagen PhD student, health economist

Tromsø, University Hospital of North Norway (UNN), Åshild Bjørnerem, PhD, gynecologist, Prof. Ragnar Joakimsen, endocrinologist, Marit Osima, MD, PhD student, Camilla Andreasen, PhD student, Jan Elvenes, PhD, Karl-Ivar Lorentzen, all orthopaedic surgeons, and Anita Kanniainen. May Greta Pedersen, nurses.

Steering Committee: Åshild Bjørnerem (project chair), Lene B Solberg (project coordinator), May-Britt Stenbro (coordinator of the project nurses), Lars Nordsletten (responsible for the budget which is located at OUS, Oslo), Tone K Omsland (responsible for data management), Trude Basso (responsible for the project web site), Frede Frihagen, Tove T Borgen, Wender Figved, Erik F Eriksen, Unni Syversen, Ellen Apalset, Cecilie Dahl and Ida Lund.

International collaborators

David Marsh, orthopaedic surgeon, UK, president Fragility Fracture Network, Kristina Åkesson, orthopaedic surgeon, Sweden, leader FLS development IOF, Stephen Gallacher, endocrinologist, Glasgow, FLS researcher and clinician, Henrik Palm, orthopaedic surgeon, Denmark, FLS researcher. They have co-authored the project application, and will advise on introduction of FLS.

2.4 Total budget for this project This is outlined in the application form.

3. Key perspectives and compliance with strategic documents

3.1 Compliance with strategic documents

This cross-regional cooperative project will strengthen service-relevant, patient-oriented clinical research and health services research within the priority area of musculoskeletal disorders as *specifically requested in the call for proposals.* A national research team is established across hospitals and universities. This project will generate new knowledge, increase competency, enhance quality and develop the health services delivered by hospitals for secondary fracture prevention. We

expect this research to provide a basis for ensuring high-quality, safe and effective services for the many fracture patients. The evidence from this project will be used to develop national guidelines for secondary fracture prevention to break the fragility fracture cycle and improve patient care.

3.2 Relevance and benefit to society

Bone fragility is currently largely ignored by treating physicians, both specialists and general practitioners. This project is a unique possibility to test the hypothesis that a new standardized program for bone fragility assessment and treatment will prevent secondary fractures and reduce mortality rates in both genders. We believe this will be useful to establish a clinical management strategy, to correctly target treatment to individuals who need it, to reduce the burden of bone fragility in the society, and promote bone health and life quality by advancing age. We wish through this research to contribute to increased understanding of the importance of bone fragility, and offer assessment and treatment to those who need it, and help focus on osteoporosis.

3.3 Environmental impact No environmental impact expected as a result of this project.

3.4 Ethical perspectives

Approval by the ethics committee has been achieved for linkage of data from national registers for the main study, with exemption from obtaining of consent (REK 2015/334 for the period 2015-2025). We will apply for access of the data from each of the national registers. The linkage of the register data will be performed by NPR, who will provide us with anonymous data that will be stored safely at TSD, at the University of Oslo (see method section).

3.5 Gender issues (Recruitment of women, gender balance and gender perspectives)

Of fracture patients 2/3 are women and 1/3 men, but men have higher mortality after a hip fracture. The nurses at each hospital will approach patients of both genders. Analyses will be gender stratified, and women and men will be compared. Moreover, the project will promote the Research Council's general objectives to increase recruitment of female collaborators and improve gender balance in projects, to obtain an equal gender distribution.

4. Dissemination and communication of results

4.1 Dissemination plan

The scientific results will be presented and published in peer-review national journals and highly renowned international journals and at local, national and international meetings. The results will also be presented for the public in local meetings, through media and in cooperation with patient

organisations. Authorship for the various scientific papers will be according to the Vancouver protocol. If in doubt about authorship, the project steering committee will decide.

4.2 Communication with users

All patients will be informed about the importance of bone fragility and advised about treatment if needed in a face-to-face setting by the coordinating nurses at each of the study sites. All information important to the participant's health and results of this research will be made available for the participants. The nurses and physicians involved in this project will educate patients, staff members and the general population, present results from successful FLS models in other countries, for prevention of secondary fractures and explain the importance of the current research project.

References

(1) Cummings SR, Melton LJ. Epidemiology and outcomes of osteoporotic fractures. Lancet 2002;359(9319):1761-7.

(2) Lofthus CM, Osnes EK, Falch JA, et al. Epidemiology of hip fractures in Oslo, Norway. Bone 2001;29(5):413-8.

(3) Seeman E. Unmet needs in fracture prevention: new European guidelines for the investigation and registration of therapeutic agents. Osteoporos Int 2007;18(5):569-73.

(4) Hernlund E, Svedbom A, Ivergard M, et al. Osteoporosis in the European Union: medical management, epidemiology and economic burden. A report prepared in collaboration with the International Osteoporosis Foundation (IOF) and the European Federation of Pharmaceutical Industry Associations (EFPIA). Arch Osteoporos 2013;8(1-2):136.

(5) Omsland TK, Magnus JH. Forecasting the burden of future postmenopausal hip fractures. Osteoporos Int 2014.

(6) Kanis JA, Johnell O, Oden A, et al. Long-term risk of osteoporotic fracture in Malmo. Osteoporos Int 2000;11(8):669-74.

(7) Kanis JA, Burlet N, Cooper C, et al. European guidance for the diagnosis and management of osteoporosis in postmenopausal women. Osteoporos Int 2008;19(4):399-428.

(8) Kanis JA, Oden A, Johnell O, et al. The components of excess mortality after hip fracture. Bone 200;32(5):468-73.

(9) Johnell O, Kanis JA, Jonsson B, et al. The burden of hospitalised fractures in Sweden. Osteoporos Int 2005;16(2):222-8.

(10) Ensrud KE, Black DM, Palermo L, et al. Treatment with alendronate prevents fractures in women at highest risk: results from the Fracture Intervention Trial. Arch Intern Med 1997;157(22):2617-24.

(11) Guyatt GH, Cranney A, Griffith L, et al. Summary of meta-analyses of therapies for postmenopausal osteoporosis and the relationship between bone density and fractures. Endocrinol Metab Clin North Am 2002;31(3):659-79, xii.

(12) Engesæter LB, Gjertsen JE, Kvinnesland I, Kvamsdal LB. The Norwegian Hip Fracture Register, annual report, The Norwegian Arthroplasty Register. 2014.

(13) Omsland TK, Holvik K, Meyer HE, et al. Hip fractures in Norway 1999-2008: time trends in total incidence and second hip fracture rates: a NOREPOS study. Eur J Epidemiol 2012;27(10):807-14.

(14) The Norwegian Institute of Public Health. Osteoporosis and fractures in Norway - fact sheet. 2014.

(15) Lofthus CM, Frihagen F, Meyer HE, et al. Epidemiology of distal forearm fractures in Oslo, Norway. Osteoporos Int 2008;19(6):781-6.

(16) Johnell O, Kanis JA. An estimate of the worldwide prevalence and disability associated with osteoporotic fractures. Osteoporos Int 2006;17(12):1726-33.

(17) Faksvåg HL. Kostnader ved hoftebrudd hos eldre. Rapport 2014. 2014(ISSN 1892-968). 2014.

(18) Borgstrom F, Sobocki P, Strom O, Jonsson B. The societal burden of osteoporosis in Sweden. Bone 2007;40(6):1602-9.

(19) Seeman E. Pathogenesis of bone fragility in women and men. Lancet 2002;359(9320):1841-50.

(20) Kannegaard PN, van der Mark S, Eiken P, Abrahamsen B. Excess mortality in men compared with women following a hip fracture. National analysis of comedications, comorbidity and survival. Age Ageing 2010;39(2):203-9.

(21) Omsland TK, Emaus N, Tell GS, et al. Mortality following the first hip fracture in Norwegian women and men (1999-2008). A NOREPOS study. Bone 2014;63:81-6.

(22) Seeman E, Delmas PD. Bone quality-the material and structural basis of bone strength and fragility. NEJM 2006;354:2250-61.

(23) Siris ES, Miller PD, Barrett-Connor E, et al. Identification and fracture outcomes of undiagnosed low bone mineral density in postmenopausal women: results from the National Osteoporosis Risk Assessment. JAMA 2001;286(22):2815-22.

(24) Schuit SC, van der KM, Weel AE, et al. Fracture incidence and association with bone mineral density in elderly men and women: the Rotterdam Study. Bone 2004;34(1):195-202.

(25) Kanis JA, Johnell O, Oden A, Johansson H, McCloskey E. FRAX and the assessment of fracture probability in men and women from the UK. Osteoporos Int 2008;19(4):385-97.

(26) Johansson H, Kanis JA, Oden A, Johnell O, McCloskey E. BMD, clinical risk factors and their combination for hip fracture prevention. Osteoporos Int 2009;20(10):1675-82.

(27) Leslie WD, Brennan SL, Lix LM, et al. Direct comparison of eight national FRAX(R) tools for fracture prediction and treatment

qualification in Canadian women. Arch Osteoporos 2013;8(1-2):145.

(28) Nguyen ND, Frost SA, Center JR, Eisman JA, Nguyen TV. Development of prognostic nomograms for individualizing 5-year and 10-year fracture risks. Osteoporos Int 2008;19(10):1431-44.

(29) Gehlbach S, Saag KG, Adachi JD, Hooven FH, Flahive J, Boonen S, et al. Previous fractures at multiple sites increase the risk for subsequent fractures: the Global Longitudinal Study of Osteoporosis in Women. J Bone Miner Res 2012;27(3):645-53.

(30) Omsland TK, Emaus N, Tell GS, et al. Ten-year risk of second hip fracture. A NOREPOS study. Bone 2013;52(1):493-7.

(31) Devold HM, Sogaard AJ, Tverdal A, Falch JA, Furu K, Meyer HE. Hip fracture and other predictors of anti-osteoporosis drug use in Norway. Osteoporos Int 2013;24(4):1225-33.

(32) Stoen RO, Nordsletten L, Meyer HE, Frihagen JF, Falch JA, Lofthus CM. Hip fracture incidence is decreasing in the high incidence area of Oslo, Norway. Osteoporos Int 2012;23(10):2527-34.

(33) Marsh D, Akesson K, Beaton DE, Bogoch ER, Boonen S, Brandi ML, et al. Coordinator-based systems for secondary prevention in fragility fracture patients. Osteoporos Int 2011;22(7):2051-65.

(34) McLellan AR, Wolowacz SE, Zimovetz EA, et al. Fracture liaison services for the evaluation and management of patients with osteoporotic fracture: a cost-effectiveness evaluation based on data collected over 8 years of service provision. Osteoporos Int 2011;22(7):2083-98.

(35) Dell R, Greene D, Schelkun SR, Williams K. Osteoporosis disease management: the role of the orthopaedic surgeon. J Bone Joint Surg Am 2008;90 Suppl 4:188-94.

(36) van Helden S, Cauberg E, Geusens P, Winkes B, van der Weijden T, Brink P. The fracture and osteoporosis outpatient clinic: an effective strategy for improving implementation of an osteoporosis guideline. J Eval Clin Pract 2007;13(5):801-5

(37) Lih A, Nandapalan H, Kim M, Yap C, Lee P, Ganda K, Seibel MJ. Targeted intervention reduces refracture rates in patients with incident non-vertebral osteoporotic fractures: a 4-year prospective controlled study. Osteoporos Int. 2011;22(3):849-58.

(38) Astrand J, Thorngren KG, Tagil M, Akesson K. 3-year follow-up of 215 fracture patients from a prospective and consecutive osteoporosis screening program. Fracture patients care! Acta Orthop 2008;79(3):404-9.

(39) Astrand J, Nilsson J, Thorngren KG. Screening for osteoporosis reduced new fracture incidence by almost half: a 6-year follow-up of 592 fracture patients from an osteoporosis screening program. Acta Orthop 2012;83(6):661-5.

(40) Haugen AS, Søfteland E, Almeland SK, Sevdalis N, Vonen B et al. Effect of the World Health Organization Checklist on Patient Outcomes: A Stepped Wedge Cluster Randomized Controlled Trial. Ann Surg. 2014. [Epub ahead of print]

(41) Mdege ND, Man MS, Taylor Nee Brown CA, Torgerson DJ. Systematic review of stepped wedge cluster randomized trials shows that design is particularly used to evaluate interventions during routine implementation. J Clin Epidemiol. 2011;64(9):936-48.
