# Supplementary material for: Effect of a Fracture Liaison Service on the Rate of Subsequent Fracture Among Patients With a Fragility Fracture in the Norwegian Capture the Fracture Initiative (NoFRACT): A Trial Protocol
Source: JAMA Netw Open. 2018 Dec 7;1(8):e185701. doi: 10.1001/jamanetworkopen.2018.5701 (PMC6324344; doi:10.1001/jamanetworkopen.2018.5701)
Supplement: Supplement 2. — Data Sharing Statement [file jamanetwopen-1-e185701-s002.pdf]

Data Sharing Statement

Andreasen. Effect of a Fracture Liaison Service on the Rate of Subsequent Fracture Among Patients With a Fragility Fracture in the Norwegian Capture the Fracture Initiative (NoFRACT). *JAMA Netw Open*. Published December 07, 2018. 10.1001/jamanetworkopen.2018.5701

Data
Data available: No

Additional Information
Explanation for why data not available: Due to strict protection of privacy under Norwegian law, individual level datasets can only be shared if approved by The Regional Committees for Medical and Health Research Ethics .
